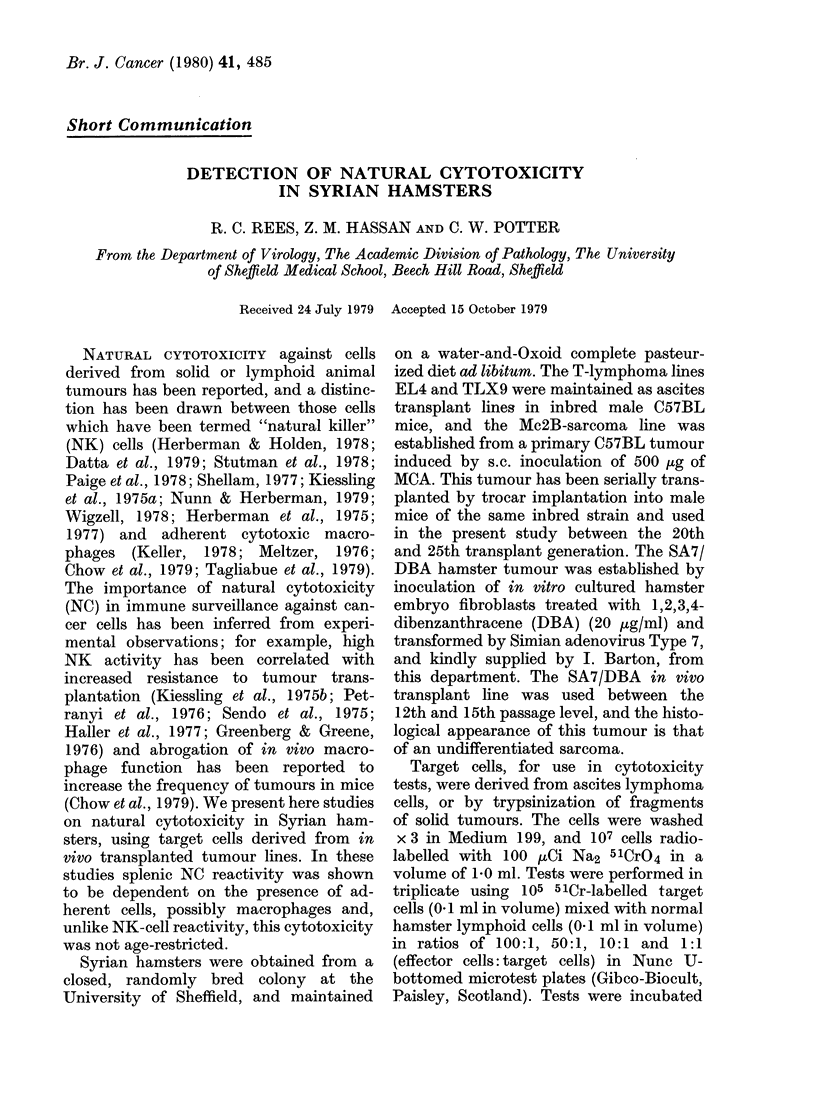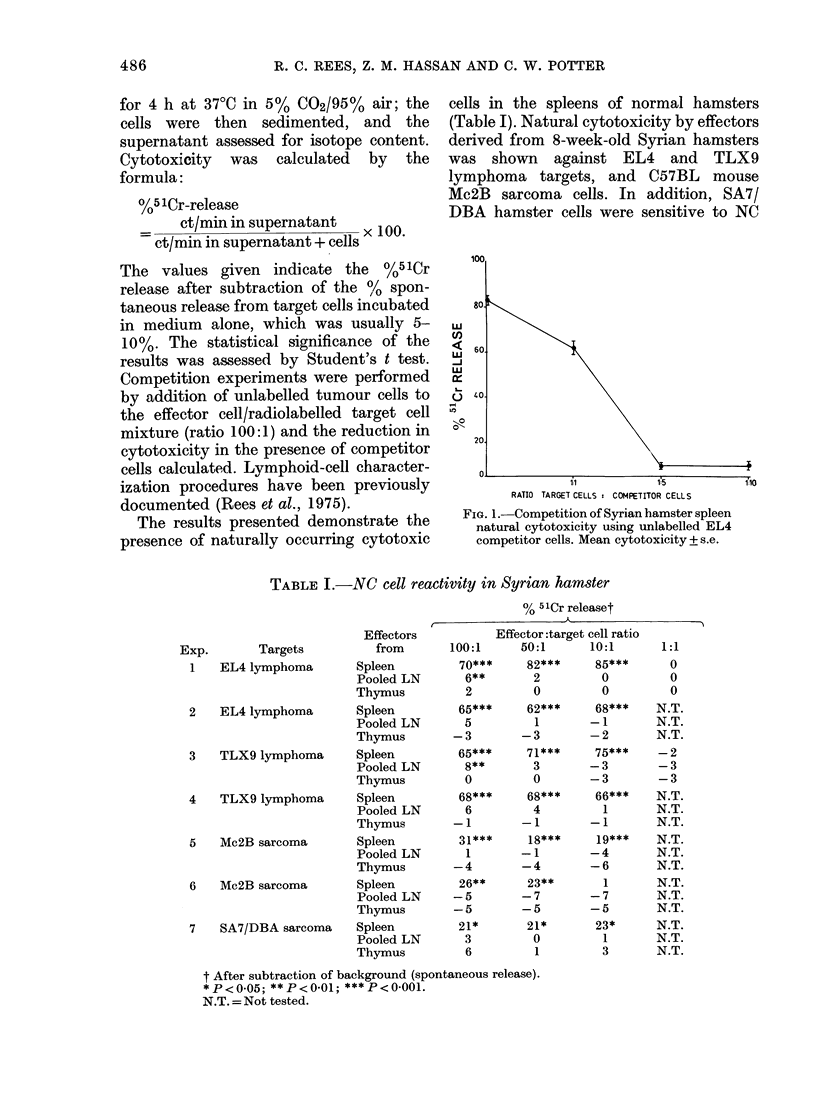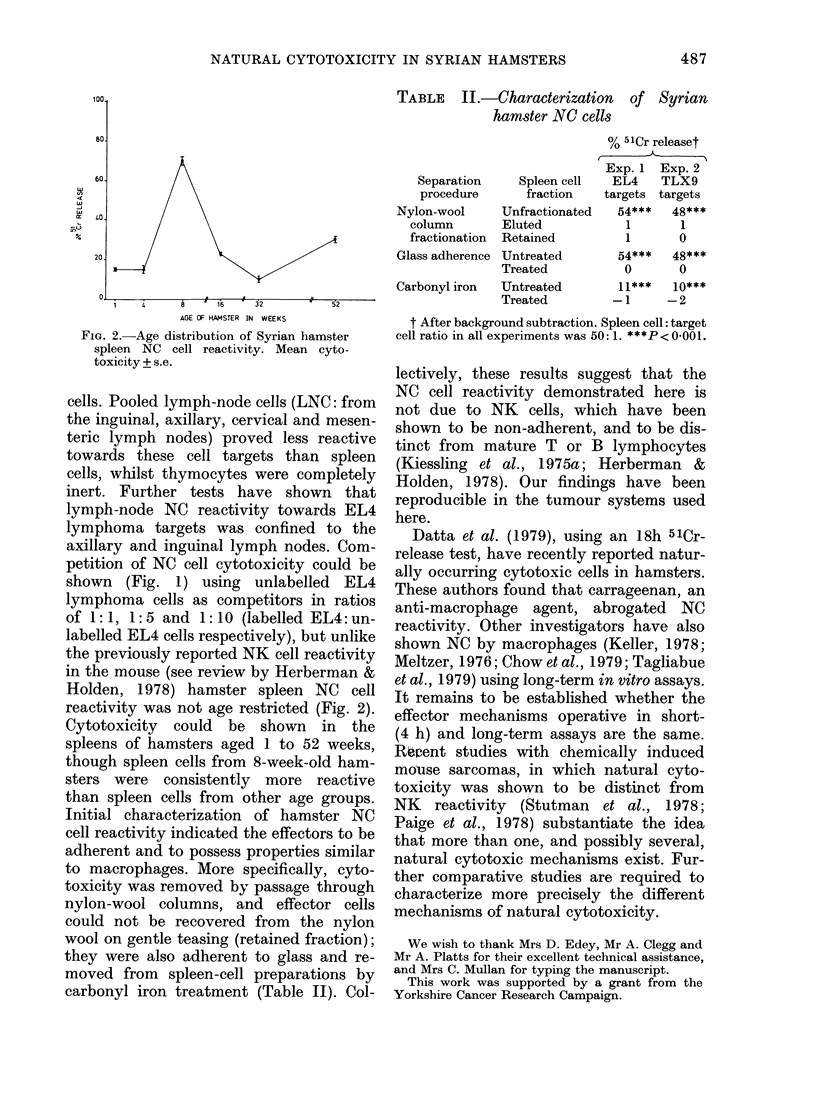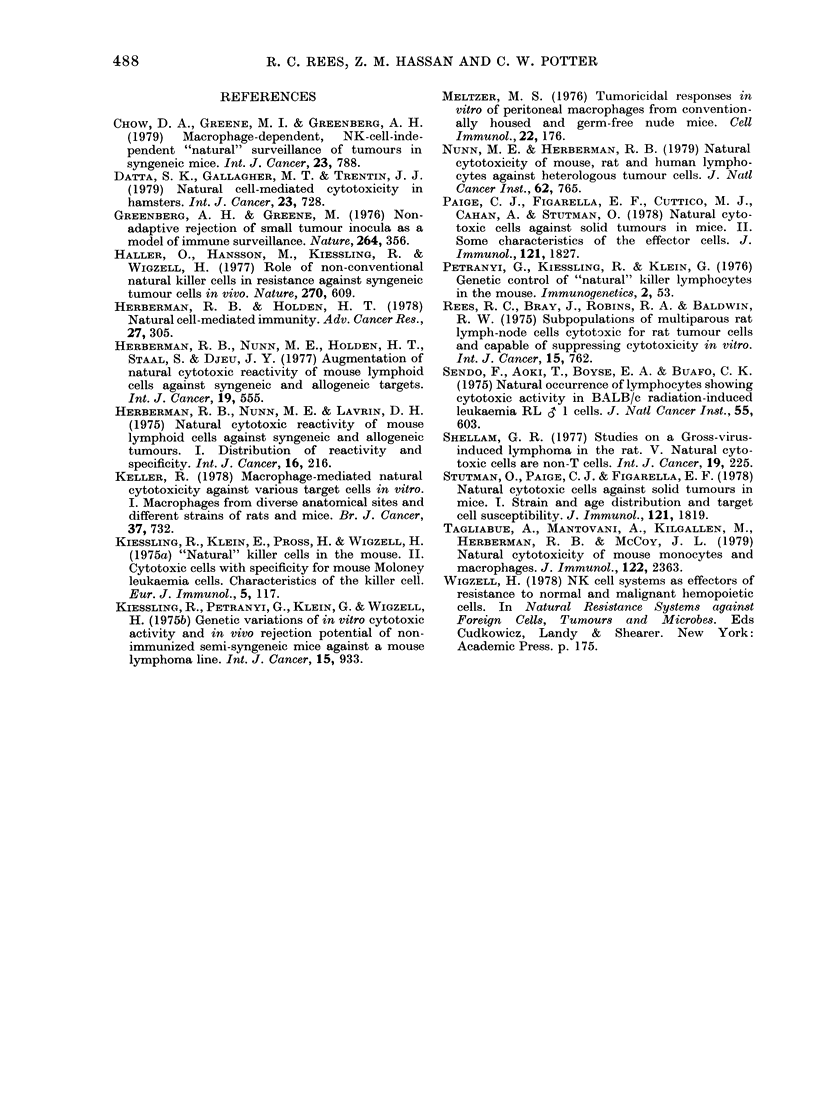# Detection of natural cytotoxicity in Syrian hamsters.

**DOI:** 10.1038/bjc.1980.75

**Published:** 1980-03

**Authors:** R. C. Rees, Z. M. Hassan, C. W. Potter


*Br. J. Cancer* (1980) **41**, 485

***Short Communication***

### DETECTION OF NATURAL CYTOTOXICITY IN SYRIAN HAMSTERS

R. C. REES, Z. M. HASSAN AND C. W. POTTER

*From the Department of Virology, The Academic Division of Pathology, The University of Sheffield Medical School, Beech Hill Road, Sheffield*

Received 24 July 1979 Accepted 15 October 1979

NATURAL CYTOTOXICITY against cells derived from solid or lymphoid animal tumours has been reported, and a distinction has been drawn between those cells which have been termed "natural killer" (NK) cells (Herberman & Holden, 1978; Datta *et al.*, 1979; Stutman *et al.*, 1978; Paige *et al.*, 1978; Shellam, 1977; Kiessling *et al.*, 1975*a*; Nunn & Herberman, 1979; Wigzell, 1978; Herberman *et al.*, 1975; 1977) and adherent cytotoxic macrophages (Keller, 1978; Meltzer, 1976; Chow *et al.*, 1979; Tagliabue *et al.*, 1979). The importance of natural cytotoxicity (NC) in immune surveillance against cancer cells has been inferred from experimental observations; for example, high NK activity has been correlated with increased resistance to tumour transplantation (Kiessling *et al.*, 1975*b*; Petranyi *et al.*, 1976; Sendo *et al.*, 1975; Haller *et al.*, 1977; Greenberg & Greene, 1976) and abrogation of *in vivo* macrophage function has been reported to increase the frequency of tumours in mice (Chow *et al.*, 1979). We present here studies on natural cytotoxicity in Syrian hamsters, using target cells derived from *in vivo* transplanted tumour lines. In these studies splenic NC reactivity was shown to be dependent on the presence of adherent cells, possibly macrophages and, unlike NK-cell reactivity, this cytotoxicity was not age-restricted.

Syrian hamsters were obtained from a closed, randomly bred colony at the University of Sheffield, and maintained on a water-and-Oxoid complete pasteurized diet *ad libitum.* The T-lymphoma lines EL4 and TLX9 were maintained as ascites transplant lines in inbred male C57BL mice, and the Mc2B-sarcoma line was established from a primary C57BL tumour induced by s.c. inoculation of 500 $\mu$g of MCA. This tumour has been serially transplanted by trocar implantation into male mice of the same inbred strain and used in the present study between the 20th and 25th transplant generation. The SA7/DBA hamster tumour was established by inoculation of *in vitro* cultured hamster embryo fibroblasts treated with 1,2,3,4-dibenzanthracene (DBA) (20 $\mu$g/ml) and transformed by Simian adenovirus Type 7, and kindly supplied by I. Barton, from this department. The SA7/DBA *in vivo* transplant line was used between the 12th and 15th passage level, and the histological appearance of this tumour is that of an undifferentiated sarcoma.

Target cells, for use in cytotoxicity tests, were derived from ascites lymphoma cells, or by trypsinization of fragments of solid tumours. The cells were washed $\times 3$ in Medium 199, and $10^7$ cells radiolabelled with 100 $\mu$Ci $Na_2\ {}^{51}CrO_4$ in a volume of 1·0 ml. Tests were performed in triplicate using $10^5$ $^{51}$Cr-labelled target cells (0·1 ml in volume) mixed with normal hamster lymphoid cells (0·1 ml in volume) in ratios of 100:1, 50:1, 10:1 and 1:1 (effector cells: target cells) in Nunc U-bottomed microtest plates (Gibco-Biocult, Paisley, Scotland). Tests were incubated for 4 h at 37°C in 5% $CO_2$/95% air; the cells were then sedimented, and the supernatant assessed for isotope content. Cytotoxicity was calculated by the formula:

$$\%^{51}\text{Cr-release} = \frac{\text{ct/min in supernatant}}{\text{ct/min in supernatant} + \text{cells}} \times 100.$$

The values given indicate the $\%^{51}$Cr release after subtraction of the % spontaneous release from target cells incubated in medium alone, which was usually 5–10%. The statistical significance of the results was assessed by Student's *t* test. Competition experiments were performed by addition of unlabelled tumour cells to the effector cell/radiolabelled target cell mixture (ratio 100:1) and the reduction in cytotoxicity in the presence of competitor cells calculated. Lymphoid-cell characterization procedures have been previously documented (Rees *et al.*, 1975).

The results presented demonstrate the presence of naturally occurring cytotoxic cells in the spleens of normal hamsters (Table I). Natural cytotoxicity by effectors derived from 8-week-old Syrian hamsters was shown against EL4 and TLX9 lymphoma targets, and C57BL mouse Mc2B sarcoma cells. In addition, SA7/DBA hamster cells were sensitive to NC

FIG. 1.—Competition of Syrian hamster spleen natural cytotoxicity using unlabelled EL4 competitor cells. Mean cytotoxicity ± s.e.

TABLE I.—*NC cell reactivity in Syrian hamster*

| | | | % $^{51}$Cr release† | | | |
|---|---|---|---|---|---|---|
| | | Effectors | Effector:target cell ratio | | | |
| Exp. | Targets | from | 100:1 | 50:1 | 10:1 | 1:1 |
| 1 | EL4 lymphoma | Spleen | 70*** | 82*** | 85*** | 0 |
| | | Pooled LN | 6** | 2 | 0 | 0 |
| | | Thymus | 2 | 0 | 0 | 0 |
| 2 | EL4 lymphoma | Spleen | 65*** | 62*** | 68*** | N.T. |
| | | Pooled LN | 5 | 1 | −1 | N.T. |
| | | Thymus | −3 | −3 | −2 | N.T. |
| 3 | TLX9 lymphoma | Spleen | 65*** | 71*** | 75*** | −2 |
| | | Pooled LN | 8** | 3 | −3 | −3 |
| | | Thymus | 0 | 0 | −3 | −3 |
| 4 | TLX9 lymphoma | Spleen | 68*** | 68*** | 66*** | N.T. |
| | | Pooled LN | 6 | 4 | 1 | N.T. |
| | | Thymus | −1 | −1 | −1 | N.T. |
| 5 | Mc2B sarcoma | Spleen | 31*** | 18*** | 19*** | N.T. |
| | | Pooled LN | 1 | −1 | −4 | N.T. |
| | | Thymus | −4 | −4 | −6 | N.T. |
| 6 | Mc2B sarcoma | Spleen | 26** | 23** | 1 | N.T. |
| | | Pooled LN | −5 | −7 | −7 | N.T. |
| | | Thymus | −5 | −5 | −5 | N.T. |
| 7 | SA7/DBA sarcoma | Spleen | 21* | 21* | 23* | N.T. |
| | | Pooled LN | 3 | 0 | 1 | N.T. |
| | | Thymus | 6 | 1 | 3 | N.T. |

† After subtraction of background (spontaneous release).
* $P < 0.05$; ** $P < 0.01$; *** $P < 0.001$.
N.T. = Not tested.

FIG. 2.—Age distribution of Syrian hamster spleen NC cell reactivity. Mean cytotoxicity ± s.e.

cells. Pooled lymph-node cells (LNC: from the inguinal, axillary, cervical and mesenteric lymph nodes) proved less reactive towards these cell targets than spleen cells, whilst thymocytes were completely inert. Further tests have shown that lymph-node NC reactivity towards EL4 lymphoma targets was confined to the axillary and inguinal lymph nodes. Competition of NC cell cytotoxicity could be shown (Fig. 1) using unlabelled EL4 lymphoma cells as competitors in ratios of 1:1, 1:5 and 1:10 (labelled EL4: unlabelled EL4 cells respectively), but unlike the previously reported NK cell reactivity in the mouse (see review by Herberman & Holden, 1978) hamster spleen NC cell reactivity was not age restricted (Fig. 2). Cytotoxicity could be shown in the spleens of hamsters aged 1 to 52 weeks, though spleen cells from 8-week-old hamsters were consistently more reactive than spleen cells from other age groups. Initial characterization of hamster NC cell reactivity indicated the effectors to be adherent and to possess properties similar to macrophages. More specifically, cytotoxicity was removed by passage through nylon-wool columns, and effector cells could not be recovered from the nylon wool on gentle teasing (retained fraction); they were also adherent to glass and removed from spleen-cell preparations by carbonyl iron treatment (Table II). Col-

TABLE II.—*Characterization of Syrian hamster NC cells*

| Separation procedure | Spleen cell fraction | % $^{51}$Cr release† Exp. 1 EL4 targets | Exp. 2 TLX9 targets |
|---|---|---|---|
| Nylon-wool column fractionation | Unfractionated | 54*** | 48*** |
| | Eluted | 1 | 1 |
| | Retained | 1 | 0 |
| Glass adherence | Untreated | 54*** | 48*** |
| | Treated | 0 | 0 |
| Carbonyl iron | Untreated | 11*** | 10*** |
| | Treated | −1 | −2 |

† After background subtraction. Spleen cell: target cell ratio in all experiments was 50:1. ***$P < 0.001$.

lectively, these results suggest that the NC cell reactivity demonstrated here is not due to NK cells, which have been shown to be non-adherent, and to be distinct from mature T or B lymphocytes (Kiessling *et al.*, 1975*a*; Herberman & Holden, 1978). Our findings have been reproducible in the tumour systems used here.

Datta *et al.* (1979), using an 18h $^{51}$Cr-release test, have recently reported naturally occurring cytotoxic cells in hamsters. These authors found that carrageenan, an anti-macrophage agent, abrogated NC reactivity. Other investigators have also shown NC by macrophages (Keller, 1978; Meltzer, 1976; Chow *et al.*, 1979; Tagliabue *et al.*, 1979) using long-term *in vitro* assays. It remains to be established whether the effector mechanisms operative in short- (4 h) and long-term assays are the same. Recent studies with chemically induced mouse sarcomas, in which natural cytotoxicity was shown to be distinct from NK reactivity (Stutman *et al.*, 1978; Paige *et al.*, 1978) substantiate the idea that more than one, and possibly several, natural cytotoxic mechanisms exist. Further comparative studies are required to characterize more precisely the different mechanisms of natural cytotoxicity.

We wish to thank Mrs D. Edey, Mr A. Clegg and Mr A. Platts for their excellent technical assistance, and Mrs C. Mullan for typing the manuscript.

This work was supported by a grant from the Yorkshire Cancer Research Campaign.

### REFERENCES

CHOW, D. A., GREENE, M. I. & GREENBERG, A. H. (1979) Macrophage-dependent, NK-cell-independent "natural" surveillance of tumours in syngeneic mice. *Int. J. Cancer*, **23**, 788.

DATTA, S. K., GALLAGHER, M. T. & TRENTIN, J. J. (1979) Natural cell-mediated cytotoxicity in hamsters. *Int. J. Cancer*, **23**, 728.

GREENBERG, A. H. & GREENE, M. (1976) Non-adaptive rejection of small tumour inocula as a model of immune surveillance. *Nature*, **264**, 356.

HALLER, O., HANSSON, M., KIESSLING, R. & WIGZELL, H. (1977) Role of non-conventional natural killer cells in resistance against syngeneic tumour cells *in vivo*. *Nature*, **270**, 609.

HERBERMAN, R. B. & HOLDEN, H. T. (1978) Natural cell-mediated immunity. *Adv. Cancer Res.*, **27**, 305.

HERBERMAN, R. B., NUNN, M. E., HOLDEN, H. T., STAAL, S. & DJEU, J. Y. (1977) Augmentation of natural cytotoxic reactivity of mouse lymphoid cells against syngeneic and allogeneic targets. *Int. J. Cancer*, **19**, 555.

HERBERMAN, R. B., NUNN, M. E. & LAVRIN, D. H. (1975) Natural cytotoxic reactivity of mouse lymphoid cells against syngeneic and allogeneic tumours. I. Distribution of reactivity and specificity. *Int. J. Cancer*, **16**, 216.

KELLER, R. (1978) Macrophage-mediated natural cytotoxicity against various target cells *in vitro*. I. Macrophages from diverse anatomical sites and different strains of rats and mice. *Br. J. Cancer*, **37**, 732.

KIESSLING, R., KLEIN, E., PROSS, H. & WIGZELL, H. (1975*a*) "Natural" killer cells in the mouse. II. Cytotoxic cells with specificity for mouse Moloney leukaemia cells. Characteristics of the killer cell. *Eur. J. Immunol.*, **5**, 117.

KIESSLING, R., PETRANYI, G., KLEIN, G. & WIGZELL, H. (1975*b*) Genetic variations of *in vitro* cytotoxic activity and *in vivo* rejection potential of non-immunized semi-syngeneic mice against a mouse lymphoma line. *Int. J. Cancer*, **15**, 933.

MELTZER, M. S. (1976) Tumoricidal responses *in vitro* of peritoneal macrophages from conventionally housed and germ-free nude mice. *Cell Immunol.*, **22**, 176.

NUNN, M. E. & HERBERMAN, R. B. (1979) Natural cytotoxicity of mouse, rat and human lymphocytes against heterologous tumour cells. *J. Natl Cancer Inst.*, **62**, 765.

PAIGE, C. J., FIGARELLA, E. F., CUTTICO, M. J., CAHAN, A. & STUTMAN, O. (1978) Natural cytotoxic cells against solid tumours in mice. II. Some characteristics of the effector cells. *J. Immunol.*, **121**, 1827.

PETRANYI, G., KIESSLING, R. & KLEIN, G. (1976) Genetic control of "natural" killer lymphocytes in the mouse. *Immunogenetics*, **2**, 53.

REES, R. C., BRAY, J., ROBINS, R. A. & BALDWIN, R. W. (1975) Subpopulations of multiparous rat lymph-node cells cytotoxic for rat tumour cells and capable of suppressing cytotoxicity *in vitro*. *Int. J. Cancer*, **15**, 762.

SENDO, F., AOKI, T., BOYSE, E. A. & BUAFO, C. K. (1975) Natural occurrence of lymphocytes showing cytotoxic activity in BALB/c radiation-induced leukaemia RL ♂ 1 cells. *J. Natl Cancer Inst.*, **55**, 603.

SHELLAM, G. R. (1977) Studies on a Gross-virus-induced lymphoma in the rat. V. Natural cytotoxic cells are non-T cells. *Int. J. Cancer*, **19**, 225.

STUTMAN, O., PAIGE, C. J. & FIGARELLA, E. F. (1978) Natural cytotoxic cells against solid tumours in mice. I. Strain and age distribution and target cell susceptibility. *J. Immunol.*, **121**, 1819.

TAGLIABUE, A., MANTOVANI, A., KILGALLEN, M., HERBERMAN, R. B. & MCCOY, J. L. (1979) Natural cytotoxicity of mouse monocytes and macrophages. *J. Immunol.*, **122**, 2363.

WIGZELL, H. (1978) NK cell systems as effectors of resistance to normal and malignant hemopoietic cells. In *Natural Resistance Systems against Foreign Cells, Tumours and Microbes*. Eds Cudkowicz, Landy & Shearer. New York: Academic Press. p. 175.